# *Candida albicans* Sfp1 Is Involved in the Cell Wall and Endoplasmic Reticulum Stress Responses Induced by Human Antimicrobial Peptide LL-37

**DOI:** 10.3390/ijms221910633

**Published:** 2021-09-30

**Authors:** Chun-Min Hsu, Yi-Ling Liao, Che-Kang Chang, Chung-Yu Lan

**Affiliations:** 1 Institute of Molecular and Cellular Biology, National Tsing Hua University, Hsinchu 30013, Taiwan; sky77531@gmail.com (C.-M.H.); ela91123@gmail.com (Y.-L.L.); steve2202543@gapp.nthu.edu.tw (C.-K.C.); 2 Department of Life Science, National Tsing Hua University, Hsinchu 30013, Taiwan

**Keywords:** *Candida albicans*, LL-37, Sfp1, cell wall, endoplasmic reticulum, unfolded protein response

## Abstract

*Candida albicans* is a commensal fungus of humans but can cause infections, particularly in immunocompromised individuals, ranging from superficial to life-threatening systemic infections. The cell wall is the outermost layer of *C. albicans* that interacts with the host environment. Moreover, antimicrobial peptides (AMPs) are important components in innate immunity and play crucial roles in host defense. Our previous studies showed that the human AMP LL-37 binds to the cell wall of *C. albicans*, alters the cell wall integrity (CWI) and affects cell adhesion of this pathogen. In this study, we aimed to further investigate the molecular mechanisms underlying the *C. albicans* response to LL-37. We found that LL-37 causes cell wall stress, activates unfolded protein response (UPR) signaling related to the endoplasmic reticulum (ER), induces ER-derived reactive oxygen species and affects protein secretion. Interestingly, the deletion of the *SFP1* gene encoding a transcription factor reduced *C. albicans* susceptibility to LL-37, which is cell wall-associated. Moreover, in the presence of LL-37, deletion of *SFP1* attenuated the UPR pathway, upregulated oxidative stress responsive (OSR) genes and affected bovine serum albumin (BSA) degradation by secreted proteases. Therefore, these findings suggested that Sfp1 positively regulates cell wall integrity and ER homeostasis upon treatment with LL-37 and shed light on pathogen-host interactions.

## 1. Introduction

*Candida albicans* is among the most common human fungal pathogens and can cause a broad range of infections, from superficial mucosal to life-threatening invasive candidiasis [1,2]. The cell wall is the outermost layer of *C. albicans* and is mainly composed of proteins and fibrillary polysaccharides [3,4,5,6,7]. Among them, mannan, β-glucan and chitin are the three major polysaccharides, and cell wall proteins are largely glycosylated with *N*- and *O*-linked mannans [5,8]. Importantly, the cell wall not only plays a critical role in maintaining cell integrity and survival but also mediates the interaction between the pathogen and host environment [4,6,7]. Therefore, cell wall components contribute to virulence-associated traits and are important targets of antifungal drugs. For example, mannans/mannoproteins mask glucans and chitins to prevent these epitopes from being recognized by the innate immune system [9,10,11]. In addition, *C. albicans* adhesion to abiotic medical devices and host cells and tissues mediated by the cell wall is essential for biofilm formation and mucosal infiltration [5,6,12]. Finally, echinocandin antifungals interfere with cell wall formation [13].

Even though the cell wall is tough, *C. albicans* dynamically alters its cell wall composition and structure to maintain its integrity in response to environmental changes and cell wall stresses [4,5,10,14,15]. One example is that caspofungin, an echinocandin, inhibits β-1,3-glucan synthesis and causes elevated chitin levels, resulting in reduced efficacy of this antifungal drug [13]. Moreover, thermal stress activates the mitogen-activated protein (MAP) kinase Mkc1, which governs the cell wall integrity signaling pathway and induces chitin reinforcement and the expression of reparative wall remodeling enzymes [16]. Additionally, LL-37 is the only member of the human cathelicidin family of antimicrobial peptides (AMPs) [17]. LL-37 is stored as a propeptide in specific neutrophil granules and is also expressed in various epithelial tissues including those of the skin, salivary glands and lungs [18,19]. Previous studies also found that LL-37 can interact with the cell surface of *C. albicans* through its binding to cell wall polysaccharides, especially mannans, as well as exoglucanase Xog1 [18,19,20,21,22]. Notably, LL-37 causes cell aggregation, cell wall remodeling and β-glucan exposure in *C. albicans* [19,20]. Consequently, LL-37 reduces *C. albicans* adhesion to plastic surfaces, oral epidermoid OECM-1 cells and urinary bladders of mice [19].

The endoplasmic reticulum (ER) is the major site for protein folding, maturation, glycosylation and secretion in eukaryotes. Upon disruption of ER homeostasis, accumulation of unfolded and misfolded proteins occurs in the ER lumen, generating a harmful condition known as ER stress [23]. To restore ER homeostasis, cells activate the unfolded protein response (UPR) signaling pathway, which is conserved among different yeast species [24,25]. In *Saccharomyces cerevisiae*, Ire1 functions as an ER stress sensor to trigger the UPR pathway [26,27]. This protein contains an N-terminal sensor domain lying inside of the ER lumen and kinase and endonuclease domains in its cytoplasmic C-terminus [26,28]. *S. cerevisiae* Ire1 normally interacts with the chaperone Kar2/BiP through its N-terminus [29,30]. Upon ER stress, Kar2/BiP dissociates from Ire1, leading to the formation of Ire1 homodimers that allow unfolded proteins to bind to the sensor domain of Ire1 [31,32]. Subsequently, Ire1 autophosphorylates and activates its endoribonuclease activity [33]. The active form of Ire1 promotes splicing of *HAC1* mRNA, which translates into the bZIP transcription factor Hac1 [34]. *S. cerevisiae* Hac1 ultimately activates the expression of UPR-responsive target genes, including those encoding protein folding-related chaperones and protein modification- and degradation-related enzymes [35]. Interestingly, complex interconnections between the yeast UPR, cell wall integrity and cellular response against stress conditions such as oxidative stress have been previously recognized [36,37].

In *C. albicans*, the functions of Ire1 have been recently characterized and found to be involved in sensing ER stress and activating the UPR pathway [38]. Intriguingly, in addition to the ER stress response, the *ire1* mutant showed additive pleiotropic effects on *C. albicans*, including antifungal tolerance, cell wall and cell membrane integrity, hyphal morphogenesis, biofilm formation and attenuated virulence, in a mouse systemic candidiasis model [38]. *C. albicans HAC1* mRNA also undergoes splicing in response to ER stress [39]. However, *C. albicans HAC1* mRNA carries a 19-bp intron that is distinct from the 250-bp intron of *S. cerevisiae HAC1* [34,39,40]. In addition, Hac1 is involved in the regulation of genes related to cell wall biosynthesis, cell surface proteins and secretory and vesicle trafficking processes and has an impact on *C. albicans* hyphal growth during ER stress [39].

*C. albicans* Sfp1 is a C2H2-type zinc finger transcription factor regulating ribosomal gene expression and carbon-conditional stress adaptation [41,42]. In addition, the *sfp1* deletion (*sfp1*Δ/*sfp1*Δ) mutant exhibits increased cell adhesion and biofilm formation and is more resistant to oxidants, macrophage-mediated killing and ROS-generating antifungals [41,43]. As mentioned above, LL-37 targets cell wall components, causes cell wall remodeling and reduces cell adhesion. Together, both LL-37 and Sfp1 seem to have common impacts on cell adhesion. Moreover, many cell adhesion molecules are cell wall components [3,5,6]. Therefore, in this study, we explored the possible relationship between Sfp1 and the cell response to LL-37. We found that LL-37 induces cell wall stress and the UPR, and Sfp1 contributes to the regulation of these stress responses.

## 2. Results

### 2.1. Deletion of SFP1 Reduces Susceptibility to LL-37

**Figure 1:**
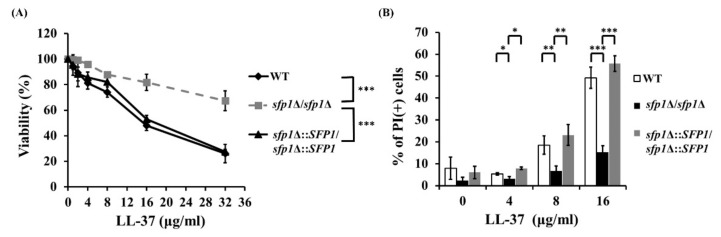
Susceptibility of *C. albicans* to LL-37. Cells were treated with or without the indicated concentrations of LL-37 at 37 °C for 30 min, and cell susceptibility was determined by CFU counting (**A**) and PI staining (**B**). The results are expressed as percentages and presented as the mean ± standard deviation (SD) of three independent experiments. WT: wild type; *sfp1*Δ/*sfp1*Δ: the *SFP1* deletion mutant; *sfp1*Δ::*SFP1*/*sfp1*Δ::*SFP1*: the *SFP1*-reintegrated strain. * *p* < 0.05; ** *p* < 0.01; *** *p* < 0.001.

To link Sfp1 and the cellular response to LL-37, the colony forming unit (CFU) counting method was used. The viability of all tested strains after LL-37 treatment decreased with increasing concentrations of the peptide. Importantly, the *sfp1*Δ/*sfp1*Δ mutant exhibited an overall higher viability with LL-37 treatment than the wild-type and *SFP1*-reintegrated strains (Figure 1A). Cell viability upon LL-37 treatment was also assessed using propidium iodide (PI) staining with flow cytometry. PI is a nonpermanent dye that can only stain dead cells with disrupted cell membranes. Figure 1B shows that the *sfp1*Δ/*sfp1*Δ mutant treated with different concentrations of LL-37 had a lower percentage of PI-positive cells than the wild-type and *SFP1*-reintegrated strains. Therefore, deletion of *SFP1* results in reduced cell susceptibility to LL-37, suggesting that Sfp1 somehow participates in the *C. albicans* response to LL-37.

### 2.2. The Effect of SFP1 Deletion on Cell Susceptibility to LL-37 Is Cell Wall-Related

**Figure 2:**
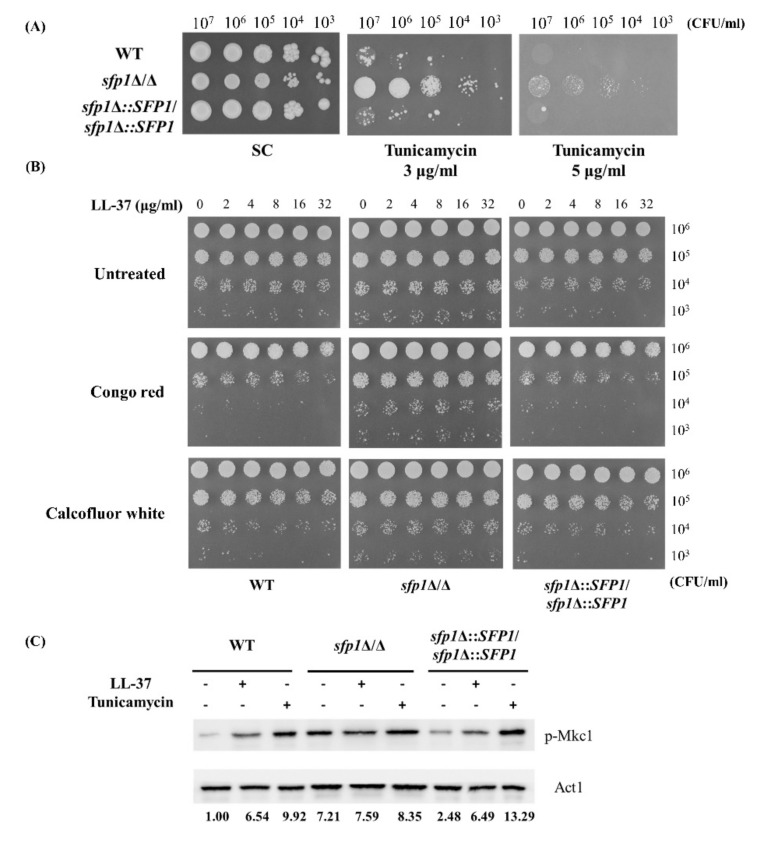
Assessment of the connection of Sfp1 with cell wall integrity and cellular response to LL-37. (**A**) Cells were serially diluted tenfold, spotted onto SC agar plates with or without tunicamycin (3 and 5 μg/mL), and incubated at 30 °C for 7 days. Representative data from at least three experiments with identified results are shown. WT: wild type; *sfp1*Δ/*sfp1*Δ: the *SFP1* deletion mutant; *sfp1*Δ::*SFP1*/*sfp1*Δ::*SFP1*: the *SFP1*-reintegrated strain. CFU: colony forming unit. (**B**) Cells were pretreated with different concentrations of LL-37 for 30 min, serially diluted tenfold and spotted onto SC agar plates with or without 30 μg/mL congo red and 180 μg/mL calcofluor white. The agar plates were incubated at 30 °C for 5 days. Representative data from at least three experiments with identified results are shown. (**C**) Mkc1 activation induced by LL-37 in different *C. albicans* strains. Cells were treated with or without LL-37 (8 μg/mL) or tunicamycin (5 μg/mL) for 30 min. Equal amounts of protein extracts from each sample were loaded. p-Mkc1 was monitored by Western blotting and analyzed with ImageJ software. The Act1 band of each sample served as the loading control and was used to normalize the phosphor-Mkc1 levels indicated by the fold change values. The data are representative of three independent experiments with identical results.

The cell wall is a dynamic structure in response to environmental changes and various stress conditions, such as the presence of human AMPs [14,15,22]. Since deletion of *SFP1* leads to reduced LL-37 susceptibility (Figure 1A,B), cell susceptibility to tunicamycin was also examined to correlate Sfp1 and the cell wall. Tunicamycin is a nucleoside antibiotic that affects the cell wall by inhibiting *N*-linked glycosylation and significantly inhibits *C. albicans* biofilm formation and maintenance [44]. As shown in Figure 2A, the *sfp1*Δ/*sfp1*Δ mutant was much more resistant to tunicamycin than the wild-type and *SFP1*-reintegrated strains.

To further determine the relationship among Sfp1, the cell wall and the cell response to LL-37, the susceptibility of cells pretreated with or without LL-37 to the cell wall-disrupting agents Calcofluor white (CFW) and Congo red (CR) and activation of the Mkc1 MAP kinase were also assessed. CFW and CR interact with chitin and β-glucan and disrupt cell wall biosynthesis [45]. In the spot assays, with treatment at different concentrations of LL-37, the *sfp1*Δ/*sfp1*Δ mutant was overall more resistant to CFW and CR than the wild-type and *SFP1*-reintegrated strains (Figure 2B). Moreover, the *C. albicans* Mkc1 signaling pathway plays an important role in cell wall construction and is activated upon cell wall stress, such as that caused by tunicamycin [46]. Tunicamycin was thus used as a positive control to detect Mkc1 activation by phosphorylation in the Western blot analysis. As expected, treatment with tunicamycin activated Mkc1 in all of the tested strains (Figure 2C). In addition, increased levels of phosphorylated Mkc1 (p-Mkc1) were detected in the wild-type and *SFP1*-reintegrated strains with LL-37 treatment compared to those without treatment. However, the *sfp1*Δ/*sfp1*Δ mutant displayed elevated levels of p-Mkc1, not only in cells treated with LL-37 but also in cells not treated (Figure 2C). A graph integrating the quantification of three independent Western blot analysis was also provided in Figure S1 (in the Supplementary Materials). Together, the results suggest that LL-37 is associated with activation of the Mkc1 signaling pathway, and the cell response to LL-37 is related to the impact of *SFP1* deletion on cell wall integrity.

### 2.3. Deletion of SFP1 Attenuates HAC1 mRNA Splicing and UPR-Responsive Gene Activation upon LL-37 Treatment

In *C. albicans*, the ER stress sensor Ire1 affects cell wall integrity, and its downstream effector Hac1 regulates the expression of genes related to cell wall biosynthesis [38,39]. Interestingly, as demonstrated in Figure 1 and Figure 2, LL-37 induces the Mkc1-mediated cell wall integrity pathway, and the impacts of Sfp1 on the cell response to LL-37 are cell wall-associated. These results thus allowed us to consider a possible correlation between the ER stress response and Sfp1 when cells were treated with LL-37.

**Figure 3:**
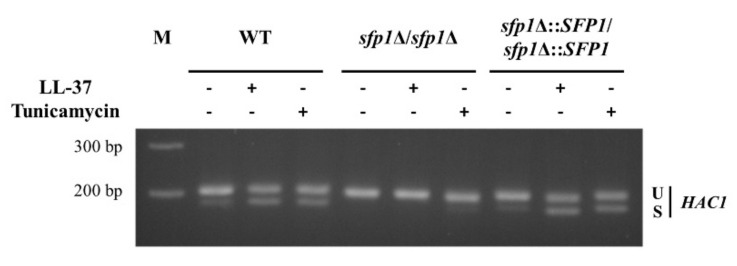
Detection of *HAC1* mRNA splicing. Cells were treated with or without LL-37 (8 μg/mL) or tunicamycin (5 μg/mL) for 30 min. Total RNA was extracted, and RT–PCR was performed. U: unspliced *HAC1*; S: spliced *HAC1*. The size difference between the unspliced and spliced cDNA is 19 bp. The data are representative of three independent experiments with identical results. M: DNA marker.

The UPR is a cellular response related to ER stress and is characterized by the splicing of *HAC1* mRNA [25]. To link the UPR and Sfp1, reverse transcription PCR was performed to detect spliced *HAC1* mRNA. In this study, cells treated with tunicamycin, which is also known to induce ER stress and activate the UPR, were included as positive controls. In Figure 3, similar to tunicamycin, LL-37 activated the UPR, which is indicated by the elevated levels of spliced *HAC1* mRNA in the wild-type and *SFP1*-reintegrated strains compared to those without LL-37 treatment. However, *HAC1* mRNA was poorly spliced not only in the *sfp*1Δ/*sfp*1Δ mutant without LL-37 treatment but also in that with treatment (Figure 3). Moreover, the levels of spliced *HAC1* mRNA in the *sfp1*Δ/*sfp1*Δ mutant appeared to be lower than those in the wild-type and *SFP1*-reintegrated strains (Figure 3). This result is somehow consistent with the finding that the *sfp1*Δ/*sfp1*Δ mutant is more resistant to tunicamycin than the control cells (Figure 2A).

**Figure 4:**
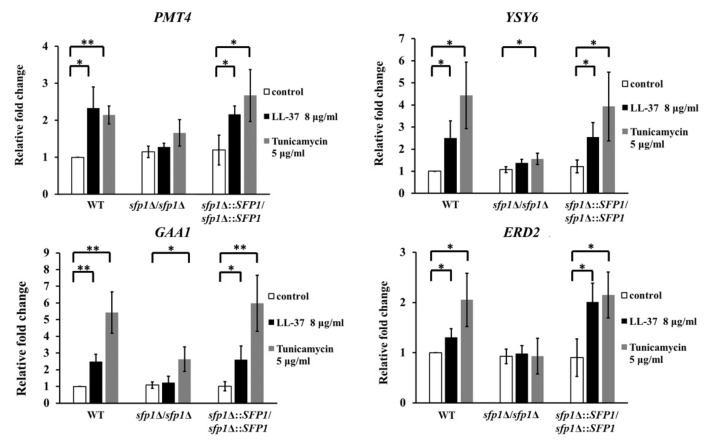
Effect of LL-37 and deletion of *SFP1* on the expression of UPR genes. Cells were treated with or without LL-37 or tunicamycin for 30 min. Real-time quantitative PCR was used to detect UPR gene expression. The *PMA1* transcripts were used as an endogenous control. The results are expressed as the mean ± SD of three independent assays. * *p* < 0.05; ** *p* < 0.01.

In response to ER stress, *C. albicans HAC1* mRNA is spliced to produce the Hac1 protein, which then activates the transcription of target genes [39]. To further connect UPR signaling and Sfp1, the expression of several UPR-responsive genes, *PMT4*, *YSY6*, *GAA1* and *ERD2* [47], under stress conditions was determined by real-time quantitative PCR (qPCR). Pmt4 is a protein mannosyltransferase, while Ysy6 is involved in the regulation of the UPR [48,49]. Moreover, Gaa1 is a putative subunit of the glycosylphosphatidylinositol (GPI)-anchor transamidase [50], and Erd2 is a protein possibly involved in secretion [51]. Transcription of these four genes was increased in the wild-type and *SFP1*-reintegrated strains with either LL-37 or tunicamycin treatment compared to that without treatment. However, the expression of these genes was not activated in the *sfp1*Δ/*sfp1*Δ mutant with LL-37 treatment compared to that without treatment (Figure 4). Together, these results indicate that LL-37 can induce ER stress and activate the UPR. In addition, deletion of *SFP1* attenuates the UPR, suggesting that Sfp1 normally contributes to the stress response to maintain ER homeostasis.

### 2.4. Deletion of SFP1 also Attenuates ROS-Induced Toxicity and Oxidation of Ero1 Promoted by LL-37

Reactive oxygen species (ROS) are natural byproducts of aerobic metabolism and critical in cell signaling and maintenance of cellular redox homeostasis [52]. However, excessive ROS accumulation can evoke cell toxicity and death. Recently, Sfp1 was found to regulate the oxidative stress response in *C. albicans* [43]. Moreover, the toxicity of cell wall stress toward *C. albicans* is mediated by ER-derived ROS [47]. Based on our findings described above, we proposed that LL-37 may alter the ER redox state and that Sfp1 is involved in this process.

**Figure 5:**
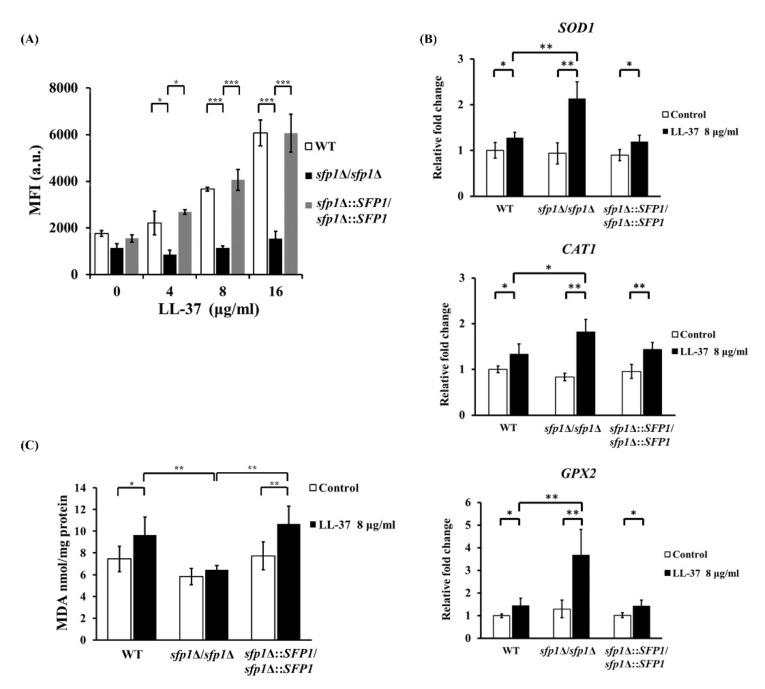
Oxidative stress in cells treated with or without LL-37. (**A**) To measure intracellular ROS accumulation, cells were treated with the indicated concentrations of LL-37 for 30 min, followed by incubation with 20 μg/mL H_2_DCFDA. The mean fluorescence of 10,000 cells was determined by flow cytometry. The results are expressed as the mean ± SD of three independent assays. * *p* < 0.05; *** *p* < 0.001. (**B**) The expression of antioxidant genes was detected using real-time quantitative PCR. The *PMA1* transcripts were used as an endogenous control. The results are expressed as the mean ± SD of three independent assays. * *p* < 0.05; ** *p* < 0.01. (**C**) Detection of lipid peroxidation as a marker of oxidative stress. Cells were treated with or without LL-37 (8 μg/mL) for 30 min, and the MDA level was measured. The results are expressed as the mean ± SD of three independent assays. * *p* < 0.05; ** *p* < 0.01.

The accumulation of intracellular ROS in the wild-type, *sfp1*Δ/*sfp1*Δ and *SFP1*-reintegrated strains with and without LL-37 treatment was compared (Figure 5A). Cells were stained with the nonpermanent ROS indicator 2′,7′-dichlorodihydrofluorescein diacetate (H_2_DCFDA), which can be cleaved by intracellular esterases to convert into the highly fluorescent 2′,7’-dichlorofluorescein (DCF). The mean fluorescence intensity of DCF was proportional to ROS levels and determined by flow cytometry. As shown in Figure 5A, the fluorescence intensity was enhanced in the wild-type and *SFP1*-reintegrated strains treated with increasing concentrations of LL-37 compared to that without treatment. However, the levels of intracellular ROS in the *sfp1*Δ/*sfp1*Δ mutant showed no significant changes among cells with or without LL-37 treatment (Figure 5A). Moreover, ROS accumulation can cause oxidative stress and activate antioxidant gene expression. To further validate the involvement of Sfp1 in the regulation of oxidative stress induced by LL-37, the expression of the oxidative stress responsive (OSR) genes *SOD1*, *CAT1* and *GPX2* was also detected. The *SOD1*, *CAT1* and *GPX2* genes encode superoxide dismutase, catalase and glutathione peroxidase, respectively [43]. As shown in Figure 5B, the expression of these antioxidant genes was upregulated in all of the tested strains with LL-37 treatment compared to that without treatment. However, the *sfp1*Δ/*sfp1*Δ mutant exhibited much higher gene activation than the wild-type and *SFP1*-reintegrated strains (Figure 5B). Finally, to link ROS accumulation and cellular toxicity in all of the tested strains, lipid peroxidation products were measured using the thiobarbituric acid reactive substance (TBARS) assay. This assay detects malondialdehyde (MDA), which is a product generated from lipid oxidation of unsaturated fatty acids, and MDA is a common marker of oxidative stress [53]. In Figure 5C, the results showed that the levels of MDA were enhanced in the wild-type and *SFP1*-reintegrated strains treated with LL-37 compared to those without treatment. However, the MDA level of the *sfp1*Δ/*sfp1*Δ mutant did not change between cells treated with and without LL-37 and was overall lower than that in the wild-type and *SFP1*-reintegrated strains upon LL-37 treatment (Figure 5C).

**Figure 6:**
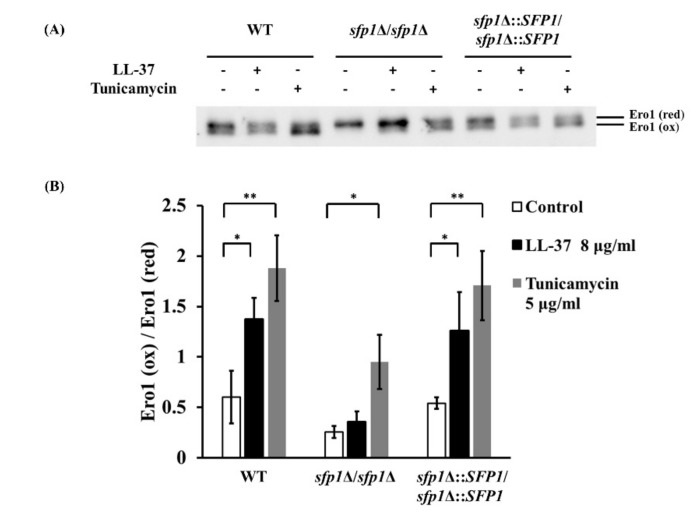
Effect of LL-37 and deletion of *SFP1* on the redox state of Ero1. (**A**) After treatment with LL-37 or tunicamycin for 30 min, total proteins were harvested. The proteins were resolved by nonreducing 6% SDS–PAGE. Western blotting was performed to detect Ero1-Flag using an anti-Flag antibody. (**B**) The ratio of Ero1(ox)/Ero1(red) was obtained by analysis using ImageJ software. The results are expressed as the mean ± SD of three independent assays. * *p* < 0.05; ** *p* < 0.01.

Ero1 is an ER-resident oxidoreductase and is important for the formation of protein disulfide bonds in the ER [47,54,55]. Moreover, the reactivation of Ero1 by molecular oxygen is one of the causes of ROS generation [56,57]. In this process, the reduced (red) form Ero1 accepts the electrons from oxygen accompanying the generation of hydrogen peroxide. Then, the oxidized (ox) form of Ero1 catalyzes oxidative protein folding through disulfide exchanges [58,59]. In other words, ER redox homeostasis is established based on a balance between Ero1 activation and inactivation [60]. Since deletion of *SFP1* diminished the effects of intracellular ROS accumulation and activation of the UPR pathway induced by LL-37 (Figure 3 and Figure 5A), these findings raise the possibility that Sfp1 affects ER redox homeostasis when cells cope with the stress induced by LL-37. To test this possibility, the redox state of Ero1 was determined using the thiol-reactive reagent 4-acetamido-4′-maleimidylstilbene-2,2′-disulfonic acid (AMS). AMS reacts with free cysteine thiols of Ero1 (red), but it cannot react with Ero1(ox) that contains no or low levels of free thiols. Therefore, after reacting with AMS, Ero1(red) should have a larger molecular weight than Ero1(ox) [47,58]. As shown in Figure 6, Western blotting was performed to detect Flag-tagged Ero1 using an anti-Flag antibody. Similar to tunicamycin treatment, LL-37 caused a 3- to 4-fold increase in the production of Ero1(ox) in both the wild-type and *SFP1*-reintegrated strains (Figure 6A,B). Conversely, there was no significant difference in the ratio of Ero1(ox)/Ero1(red) in the *sfp1*Δ/*sfp1*Δ mutant with LL-37 treatment compared to that without treatment (Figure 6).

In summary, LL-37 induces intracellular ROS accumulation, causes lipid peroxidation, an indicator of oxidative damage to cells, and enhances Ero1 oxidation. These results indicate that ER homeostasis is affected by LL-37. Moreover, these effects were observed in the wild-type and *SFP1*-reintegrated strains but not in the *sfp1*Δ/*sfp1*Δ mutant. Our findings further suggest that Sfp1 normally participates in the stress response to maintain ER homeostasis.

### 2.5. Deletion of SFP1 Affects Protein Secretion and BSA Degradation in Cells Treated with LL-37

In the secretory pathway of *C. albicans*, proteins are translocated to the ER for a variety of processes, such as folding and glycosylation. Following movement through the Golgi and secretory vesicles, proteins are further transported to various destinations, including the cell wall or the cell exterior [61]. Therefore, the UPR is essential not only for controlling protein quality but also for maintaining normal secretory function [62].

**Figure 7:**
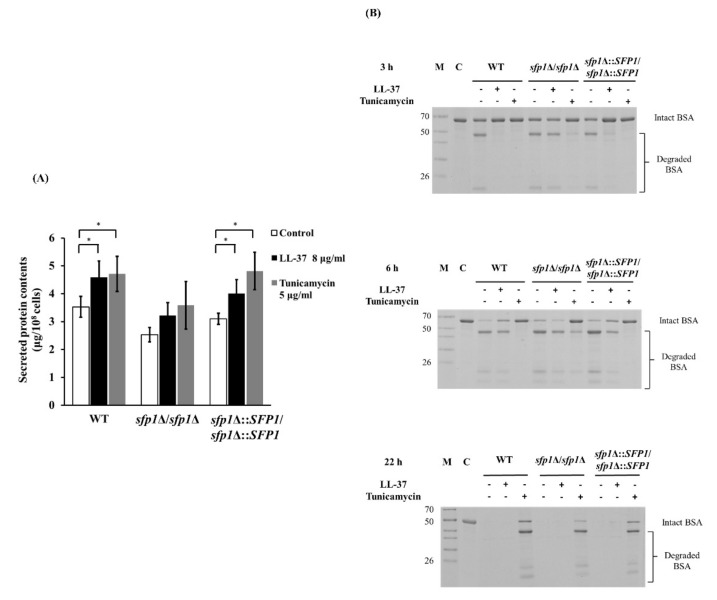
Effect of LL-37 and deletion of *SFP1* on protein secretion and functional efficiency of secreted proteases. (**A**) After treatment with or without LL-37 and tunicamycin for 30 min, proteins in the supernatant were collected and quantified using the Bradford protein assay. The results are expressed as the mean ± SD of three independent assays. * *p* < 0.05. (**B**) To determine the efficiency of secreted proteases, BSA degradation was assessed. Cells pretreated with or without LL-37 (8 μg/mL) and tunicamycin (5 μg/mL) were incubated with BSA for 3, 6 and 22 h at 30 °C. Equal amounts of cell suspension were analyzed by 12% SDS–PAGE, and proteins were visualized by Coomassie blue staining. M: protein marker ladder (kDa); C: control (BSA alone). The data are representative of three independent experiments with identical results.

Since Sfp1 is involved in the UPR induced by LL-37 (Figure 3 and Figure 4), we were thus interested in further determining the impacts of LL-37 and deletion of *SFP1* on extracellular secreted protein. In this study, the levels of secreted proteins in the supernatant were quantified from cells treated with or without LL-37. In Figure 7A, the wild-type and *SFP1*-reintegrated strains showed elevated levels of total secreted protein after LL-37 treatment compared to those without treatment. Increased levels of secreted protein were also found in cells treated with the ER stress inducer tunicamycin (Figure 7A), suggesting that LL-37 has an impact on the levels of secreted protein. However, no statistically significant difference was detected between the *sfp1*Δ/*sfp1*Δ mutant cells with or without LL-37 treatment (Figure 7A). Moreover, the *sfp1*Δ/*sfp1*Δ mutant had an overall lower secreted protein than the wild-type and *SFP1*-reintegrated strains. This result suggests that Sfp1 normally contributes to the levels of secreted protein.

Many secreted and cell wall components are virulence-associated in *C. albicans*. For example, secreted aspartyl proteases (Saps) help *C. albicans* degrade host proteins [63]. To further determine whether LL-37 and deletion of *SFP1* can alter the functional efficiency of secreted proteases, a bovine serum albumin (BSA) degradation assay was performed as previously described [64,65]. As a control for ER stress, cells treated with tunicamycin were also used. Almost no BSA degradation was observed subsequent to tunicamycin treatment for 3 and 6 h, and BSA degradation was still incomplete even after treatment for 22 h (Figure 7B). In addition, BSA was slowly degraded in the wild-type and *SFP1*-reintegrated strains, in which BSA degradation began to be detected following 6 h of incubation with LL-37 (Figure 7B). More interestingly, the *sfp1*Δ/*sfp1*Δmutant degraded BSA much more rapidly than the wild-type and *SFP1*-reintegrated strains, beginning to be observed following 3 h of incubation with LL-37 (Figure 7B). Together, this result indicates that LL-37 and Sfp1 somehow affect the efficiency of secreted proteases in BSA degradation.

## 3. Discussion

*C. albicans* encounters various environmental stresses within the host. Since the cell wall is the initial site for *C. albicans* to interact with its external environment, the structural and functional integrity of the cell wall is therefore critical for cell survival under stress conditions. For example, our previous studies showed that LL-37 associates with cell wall components of *C. albicans* and causes cell wall remodeling [19,20,21,22]. Moreover, many signaling pathways and transcriptional regulatory machinery have been identified to participate in the maintenance of cell wall integrity in response to environmental stresses [16,66,67]. One example is the Mkc1 MAP kinase signaling cascade that relates to the cell wall integrity of *C. albicans* and is activated under different stress conditions, such as that caused by cell wall-disturbing agents [68,69]. In addition, the transcription factors Cas5, Sko1 and Rlm1 regulate the cellular response to cell wall stress caused by caspofungin [66]. In this study, we expanded the current understanding of the environmental stress response of *C. albicans* relevant to the maintenance of cell wall integrity. In particular, the role of the transcription factor Sfp1 in the stress response induced by LL-37 was characterized. As determined by CFU counting, PI staining, cell susceptibility to cell wall-disturbing agents and Western blotting for Mkc1 phosphorylation, the results suggested that the impact of Sfp1 on the stress response to LL-37 is cell wall-related (Figure 1A,B and Figure 2A–C).

The significance of ER homeostasis for maintaining cell wall integrity has been reported in different fungal species. Moreover, the UPR is important for maintaining ER homeostasis [24,25]. In *S. cerevisiae*, cell wall stress activates the UPR through the Mkc1 MAP kinase signaling cascade, and mutants lacking a functional UPR are defective in cell wall biosynthesis and hypersensitive to cell wall-targeted antifungals [37,68]. Similarly, a link between cell wall stress, ER function and ER-derived ROS generation was revealed in *C. albicans* [47]. In addition, suppressed expression of *C. glabrata KRE5*, a gene encoding the ER-resident UDP-glucose: glycoprotein glucosyltransferase, significantly alters the levels of cell wall carbohydrates, induces ER stress-related gene expression and activates phosphorylation of Slt2, the homolog of *C. albicans* Mkc1 [70]. Finally, the UPR is also involved in fungal virulence. Mutants lacking HacA in *Aspergillus fumigatus* and Hxl1 in *Cryptococcus neoformans*, the homologs of Hac1, attenuate virulence in mouse models of infection [71,72]. LL-37 is a multifunctional AMP commonly found at mucosal surfaces of humans and plays an important role in the first line of defense against microbial infection [17,73]. Our previous studies showed that LL-37 targets the cell wall and affects *C. albicans* adhesion to abiotic and biotic surfaces, including oral epidermal cells and mouse bladder mucosa [19,22]. For the results reported herein, we further demonstrated that LL-37 can induce the UPR pathway. To our knowledge, this study is the first to show that the UPR is required for maintaining the cell wall integrity of a fungal pathogen to prevent damage from human AMP.

Of special interest in this study is that Sfp1 contributes to signaling and regulation of the cell response to LL-37. Sfp1 was previously found to be involved in the regulation of genes related to ribosomal protein (RP), ribosome biogenesis (Ribi), cell wall adhesins and antioxidants [41,43]. Moreover, the *SFP1* gene is activated under stress conditions during cell growth on glucose, and Sfp1 controls stress genes in a carbon source-conditional manner [42]. Consequently, Sfp1 modulates cell adhesion, biofilm formation, oxidative stress response and carbon source-conditional stress adaptation. In the present study, deletion of *SFP1* attenuated *HAC1* mRNA splicing, UPR gene activation, ROS accumulation and Ero1 oxidation promoted by LL-37 (Figure 3, Figure 4, Figure 5 and Figure 6). Finally, deletion of *SFP1* also affected the secretion and functional efficiency of extracellular proteins in cells treated with LL-37 (Figure 7A,B). The results displayed here suggest that Sfp1 is also critical for maintaining ER homeostasis. Together, Sfp1 possesses global regulatory functions and plays important roles in a wide range of stress responses in *C. albicans*.

Even though this study provides some new insights into the adaptive stress response of *C. albicans*, many questions are still worth addressing. For example, LL-37 changes the redox state of Ero1 to generate ER-derived ROS. However, mitochondria are another major site of ROS generation. The possible effects of LL-37 on mitochondrial function and mitochondrial ROS generation and the participation of Sfp1 in these effects remain unclear. Moreover, the relationship among ER stress, cell wall integrity and various signaling cascades has been reported in different fungi. These signaling cascades are exemplified by the target of rapamycin (TOR) and calcium/calcineurin pathways [74,75,76]. In this study, we indicated that the Mkc1 MAPK pathway is involved in the Sfp1-regulated cell response to LL-37 (Figure 2C). Interestingly, our previous study also showed that Sfp1 functions downstream of the small GTPase Rhb1-TOR pathway in the regulation of cell adhesion and biofilm formation [41]. Moreover, Rhb1 also regulates expression of secreted aspartyl protease 2 (Sap2) through the TOR pathway [65]. Therefore, it will be interesting to further explore the involvement of the Rhb1, TOR and calcium/calcineurin pathways in Sfp1 regulation of the LL-37 stress response.

**Figure 8:**
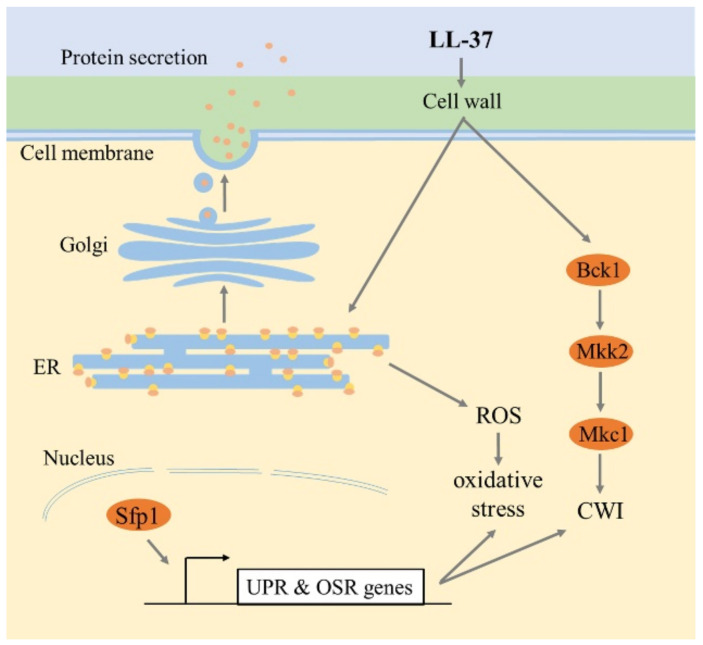
An overview of the role of Sfp1 in the cellular stress response induced by LL-37. Based on the findings of previous studies and this study, LL-37 targets cell wall components and causes cell wall remodeling. Consequently, LL-37 activates the Mkc1 MAP kinase signaling pathway to maintain cell wall integrity (CWI). LL-37 also triggers the UPR pathway to adapt and respond to ER stress conditions. Moreover, LL-37 likely induces ER-derived ROS accumulation to cause oxidative stress and affects ER-related protein secretion. Interestingly, analyses of deletion of the *SFP1* gene further correlated the role of the transcription factor Sfp1 in cell wall maintenance, ER homeostasis and oxidative stress response upon LL-37 treatment. OSR genes: oxidative stress responsive genes.

In conclusion, our analysis has revealed a new function of Sfp1 in *C. albicans*. As outlined in Figure 8, Sfp1 normally contributes to the maintenance of cell wall integrity and ER homeostasis under stress conditions such as that induced by LL-37. Ultimately, this study has highlighted the complexity of signaling and transcriptional networks in the interplay between fungal pathogens and AMPs in host innate immunity.

## 4. Materials and Methods

### 4.1. Peptide and Reagents

LL-37 (LLGDFFRKSKEKIGKEFKRIVQRIKDFLRNLVPRTES) was synthesized by MDBio, Inc. (Taipei, Taiwan). The peptide was >95% pure, as verified by high-performance liquid chromatography (HPLC) and mass spectrometry. All reagents were obtained from Sigma–Aldrich unless indicated otherwise.

### 4.2. C. albicans Strains and Growth Conditions

The *C. albicans* strains used in this study are listed in Table S1 (in the Supplementary Materials). The cells were routinely grown in YPD medium (1% yeast extract, 2% peptone and 2% glucose) and synthetic complete (SC) medium (0.67% yeast nitrogen base [YNB] with ammonium sulfate, 2% glucose and 0.079% complete supplement mixture). Plates were prepared with 1.5% agar. One colony was inoculated into YPD broth and incubated at 30 °C overnight (~16 h) with shaking at 180 rpm. This culture was harvested by centrifugation, washed twice with sterile phosphate-buffered saline (PBS), subcultured in SC medium (with an initial concentration ~1.5 × 10^7^ cells/mL) and grown to the exponential phase for further experiments. For the induction of the *MAL2* promoter and BSA degradation assay, YPM medium (1.0% yeast extract, 2.0% meat peptone and 2% maltose) and YCB-BSA-YE medium (1.17% Difco yeast carbon base, 0.01% BSA, 0.001% yeast extract, adjusted to pH 4.4 with HCl) were used separately as previously described [77].

### 4.3. Cell Susceptibility to LL-37 and Other Agents

Cell susceptibility to LL-37 was examined by CFU counting and PI staining. Briefly, *C. albicans* cells (~3.0 × 10^7^ cells/mL) were resuspended in LYM medium [78] containing different concentrations of LL-37 and incubated at 37 °C with slow shaking (100 rpm) for 30 min. Subsequently, cells were grown on YPD agar plates and incubated at 30 °C for 48 h, and the number of CFUs was counted. For PI staining, cells were treated with or without different concentrations of LL-37 for 30 min as described above and collected by centrifugation. After washing twice with PBS, cells were resuspended in PBS containing 5 μg/mL PI and incubated at room temperature for 5 min [43]. The fluorescence intensity of PI-positive cells was measured using an Accuri C6 flow cytometer (BD Biosciences, San Jose, CA, USA) with the FL2 filter. Cells without LL-37 treatment were used as negative controls. The average fluorescence intensity from at least three experiments was determined.

Cell susceptibility to cell wall-disrupting agents, CR and CFW, and the ER stress inducer tunicamycin was determined using a spot assay. Cells were treated with or without different concentrations of LL-37 and incubated at 37 °C for 30 min. Subsequently, cells were collected by centrifugation, washed twice with PBS and resuspended in sterile double-distilled water (ddH_2_O). In this case, 10 microliters of each 10-fold serially diluted *C. albicans* cells were spotted onto SC agar plates containing CR (30 μg/mL), CFW (180 μg/mL) or different concentrations of tunicamycin. Cell viability was recorded after incubation at 30 °C for 1–7 days.

### 4.4. Assay for Mkc1 Phosphorylation

Protein extraction was performed as previously described [79,80] with some modifications. Briefly, cells were treated with or without LL-37 (8 μg/mL) and tunicamycin (5 μg/mL) for 30 min, harvested and washed twice with PBS. The cell pellets were suspended in lysis buffer (50 mM HEPES [pH 7.4], 5 mM ethylenediaminetetraacetic acid [EDTA], 1% Triton X-100, 1 mM NaF, 1 mM Na_3_VO_4_, 0.2 mM phenylmethylsulfonyl fluoride [PMSF], 4 μM leupeptin, 1 μM pepstatin A and 1 μg/mL aportinin) with acid-washed glass beads. The mixture was vortexed for 30 s and placed on ice for 30 s, and this process was repeated 10 times. Insoluble material was removed by centrifugation at 4 °C at 12,000× *g* for 10 min, and the supernatant of soluble proteins was collected. Protein concentration was measured using the Bradford Protein Assay (Bio–Rad, Hercules, CA, USA).

For Western blotting to detect phosphorylation of Mkc1, 30 μg of protein was mixed with 4x sample buffer (250 mM Tris-HCl [pH 6.8], 40% glycerol, 10% β-mercaptoethanol, 8% sodium dodecyl sulfate [SDS] and 0.4% bromophenol blue), boiled for 5 min and placed on ice for 5 min. Each protein sample was then separated by 10% SDS–PAGE and transferred onto a polyvinylidene difluoride (PVDF) membrane. The membrane was blocked overnight with 5% nonfat milk at room temperature. Anti-phospho-p44/p42 MAPK antibody (#4370; Cell Signaling Technology, Danvers, MA, USA) and anti-β-actin antibody (GTX109639; GeneTex, Hsinchu, Taiwan) were used to detect phosphor-Mkc1 and Act1, respectively. Horseradish peroxidase (HRP)-conjugated goat anti-rabbit IgG (GTX213110–01; GeneTex) was used as the secondary antibody. The blots were visualized using enhanced chemiluminescence reagents (PerkinElmer Inc., Waltham, MA, USA) and an ImageQuant LAS 4000 mini biomolecular imager (GE Health care, Chicago, IL, USA). Anti-β-actin was used as a loading control, and the bands were analyzed using ImageJ software [81].

### 4.5. Assay for HAC1 mRNA Splicing

To examine splicing of *C. albicans HAC1* mRNA, cells were treated with or without LL-37 (8 μg/mL) and tunicamycin (5 μg/mL) at 37 °C for 30 min, harvested by centrifugation and washed with ice-cold ddH_2_O. Total RNA extraction and cDNA synthesis by reverse transcription were performed as previously described [82]. To detect spliced and unspliced *HAC*1 mRNA, cDNA was used as a template in a PCR as follows: 95 °C for 5 min; 30 cycles consisting of 30 s at 95 °C, 30 s at 55 °C and 30 s at 72 °C; and 10 min at 72 °C [83]. The primers used are listed in Table S2 (in the Supplementary Materials). The PCR products of spliced and unspliced *HAC1* mRNA were 189 and 208 bp, respectively, and separated by a 3% DNA agarose gel.

### 4.6. Real-Time Quantitative PCR (qPCR)

For real-time qPCR to detect UPR gene expression, the StepOne Plus™ real-time PCR system (Applied Biosystems, Framingham, MA, USA) was used. Each 15-μL reaction mixture contained 30 ng cDNA, 300 nM each forward and reverse primer, and 7.5 μL Power SYBR green PCR master mixture (Applied Biosystems). The primers used are listed in Table S2. The reactions were performed with 1 cycle at 95 °C for 10 min, followed by 40 repeated cycles at 95 °C for 15 s and 60 °C for 1 min. The *PMA1* transcripts were used as an endogenous control for qPCR [83]. All experiments were repeated independently at least three times, and the average C_T_ values were determined. The relative fold change in the expression of each gene was calculated using the 2^–ΔΔ*C*T^ method [84].

### 4.7. Measurement of Intracellular Reactive Oxygen Species (ROS)

To monitor intracellular ROS accumulation, the cell-permeant reagent H_2_DCFDA was used as a probe for measuring ROS. H_2_DCFDA is cleaved by intracellular esterase to become DCFH, which is later oxidized by ROS to form fluorescent DCF [85]. Briefly, cells treated with or without different concentrations of LL-37 were harvested by centrifugation and washed twice with sterile PBS. The cells were then incubated with 20 μg/mL H_2_DCFDA at 30 °C for 30 min. The cells were washed three times with ice-cold PBS, and the fluorescence intensity was measured using a flow cytometer (BD Accuri^®^ C6, BD Biosciences) with the FL1 filter.

### 4.8. Lipid Peroxidation Assay

To determine the extent of lipid peroxidation, the levels of MDA were measured. *C. albicans* cells were treated with or without LL-37 (8 μg/mL) for 30 min. Subsequently, cells were harvested, washed twice with PBS and resuspended in PBS containing acid-washed glass beads. The cells were vortexed for 30 s and cooled on ice for 30 s, and this process was repeated 10 times. Cell debris was removed by centrifugation at 4 °C for 10 min, and the supernatant was collected. Next, lipid peroxidation was measured using the TBARS (TCA method) Assay Kit (Cayman Chemical, Ann Arbor, MI, USA) according to the manufacturer’s instructions. In this assay, TBARS formed by the reaction of MDA and thiobarbituric acid (TBA) under high temperature (90–100°C) and acidic conditions was measured colorimetrically at 530 nm.

### 4.9. Construction of the Ero1-Flag Expressing Strains

To construct the Ero1-Flag-expressing strains, the DNA sequence of Flag-tag was independently fused to one allele of the *ERO1*-coding sequence (at its 3′-end) in the wild-type (WT), *sfp1*Δ/*sfp1*Δ and *SFP1*-reintegrated strains. The strains generated were designated WT+Ero1-Flag, *sfp1*Δ/*sfp1*Δ+Ero1-Flag and *SFP1*-reintegrated+Ero1-Flag.

For strain construction, the *SAT1*-flipper method was used [77]. A KpnI-XhoI DNA fragment composed of the 3′-end of *ERO1* was PCR-amplified from the SC5314 genome with the primer pair ERO1UR-F-KpnI and ERO1UR-R-Flag-XhoI (Table S2). Similarly, another SacII-SacI DNA fragment composed of the 3′ flanking region of *ERO1* was amplified from the SC5314 genome with the primer pair ERO1DR-F-SacII and ERO1DR-R-SacI (Table S2). The resulting DNA fragments were independently cloned into the pSFS2A vector [77] to generate pSFS2A-ERO1-Flag. A large DNA fragment carrying the *SAT1*-flipper cassette flanked by the two PCR-amplified DNA fragments was then excised from pSFS2A-ERO1-Flag by KpnI/SacI digestion. The linear DNA fragment was purified and transformed into *C. albicans* cells for integration into the chromosome via homologous recombination. The transformants were selected for nourseothricin (Nou) resistance and verified by PCR. To pop out the *SAT1*-flipper cassette, cells were grown overnight at 30 °C in 5 mL of YPM medium to induce *MAL2* promoter-regulated recombinase. Overnight-cultured cells were plated on YPM agar plates and incubated at 30 °C for 2 days. Single colonies were then screened for Nou resistance by streaking them on both YPD and YPDNou plates, and the Nou-sensitive strains were picked for PCR to verify the pop out of the *SAT1*-flipper cassette.

### 4.10. Detection of the Redox State of Ero1

To assess the redox state of Ero1, cells were treated with or without LL-37 (8 μg/mL) and tunicamycin (5 μg/mL) for 30 min, harvested by centrifugation, and washed twice with PBS. Protein extraction was performed as described above, and the protein concentration was measured using the Bradford Protein Assay (Bio–Rad, Hercules, CA, USA).

Proteins were mixed with 4x nonreducing sample buffer (250 mM Tris-HCl [pH 6.8], 40% glycerol, 8% SDS and 0.4% bromophenol blue) containing 25 mM 4-acetamido-4′-maleimidylstilbene-2,2′-disulfonic acid (AMS) (A485; Thermo Fisher Scientific, Waltham, MA, USA). The mixture was placed on ice for 15 min, incubated at 37 °C for 60 min, and boiled for 2 min [47,56,86]. The samples were resolved by nonreducing 6% SDS–PAGE and transferred to a PVDF membrane. The proteins were probed with an anti-Flag mouse monoclonal antibody (F3165, Sigma–Aldrich). HRP-conjugated goat anti-mouse IgG (GTX213111–01; GeneTex) was used as the secondary antibody. The ratio of oxidative Ero1 to reductive Ero1 was analyzed using ImageJ software [81].

### 4.11. Protein Secretion and BSA Degradation Assay

To determine protein secretion, the total content of extracellular proteins was assessed as previously described [47]. Cells were treated with or without LL-37 (8 μg/mL) and tunicamycin (5 μg/mL) for 30 min. Subsequently, the cells were centrifuged at 12,000× *g* for 10 min to collect the supernatants. Afterward, the supernatants were ultrafiltered with an Amicon Ultra4 Centrifugal Filter Unit (Millipore, Burlington, MA, USA) to concentrate the samples, and protein concentrations were determined using the Bradford Protein Assay (Bio–Rad).

The function of secreted protein was also evaluated by BSA degradation as previously described [64,65]. *C. albicans* cells grown overnight were harvested by centrifugation and washed twice with PBS. Cells were then subcultured in YCB-BSA-YE medium containing LL-37 (8 μg/mL) or tunicamycin (5 μg/mL) and incubated at 30 °C. After centrifugation at 12,000× *g* for 10 min, the supernatant protein samples were collected, separated by 12% SDS–PAGE and visualized with Coomassie blue staining.

### 4.12. Statistical Analysis

Differences in each sample were compared by the two-tailed Student’s *t*-test. *p* values < 0.05 were considered to be statistically significant.

## Data Availability

The data presented in this study are available within the article and the Supplementary Materials.

## Supplementary Materials

The following are available online at https://www.mdpi.com/article/10.3390/ijms221910633/s1.
