# Supplementary material for: Candida albicans Sfp1 Is Involved in the Cell Wall and Endoplasmic Reticulum Stress Responses Induced by Human Antimicrobial Peptide LL-37
Source: Int J Mol Sci. 2021 Sep 30;22(19):10633. doi: 10.3390/ijms221910633 (PMC8508991; doi:10.3390/ijms221910633)
Supplement: Supplementary file 1 [file ijms-22-10633-s001.zip › Proofreading Supplement final.pdf]

## Supplementary Materials

### Table S1. Strains used in this study

| Strain | Genotype | Background | #Source |
|---|---|---|---|
| SC5314 | Wild type | | [1] |
| *sfp1*Δ/*sfp1*Δ | *sfp1*Δ::*FRT*/*sfp1*Δ::*FRT* | SC5314 | [2] |
| *SFP1*RD | *sfp1*Δ::*SFP1-FRT*/*sfp1*Δ::*SFP1-FRT* | SC5314 | [2] |
| WT+Ero1-Flag | *ERO1/ERO1-FLAG* | SC5314 | This study |
| *sfp1*Δ/Δ+Ero1-Flag | *sfp1*Δ::*FRT*/*sfp1*Δ::*FRT* *ERO1/ERO1-FLAG* | *sfp1*Δ/*sfp1*Δ | This study |
| *SFP1*RD+Ero1-Flag | *sfp1*Δ::*SFP1-FRT*/*sfp1*Δ::*SFP1-FRT* *ERO1/ERO1-FLAG* | *SFP1*RD | This study |

### #Source


1. Gillum, A.M.; Tsay, E.Y.H.; Kirsch, D.R. Isolation of the *Candida albicans* gene for orotidine-5′-phosphate decarboxylase by complementation of *S. cerevisiae* ura3 and *E. coli* pyrF mutations. *Mol. Gen. Genet.* **1984**, *198*, 179–182, doi:10.1007/BF00328721.
2. Chen, H.F.; Lan, C.Y. Role of *SFP1* in the regulation of *Candida albicans* biofilm formation. *PLoS ONE* **2015**, *10*, e0129903, doi:10.1371/journal.pone.0129903.

**Table S2. Oligonucleotides used in this study**

| **Primer name** | **Sequence (5' to 3')[a]** |
|---|---|
| ERO1UR-F-KpnI | CCGCGGTACCAAGAATTGAAATTGTTGCCTTGA |
| ERO1UR-R-Flag-XhoI | GGCGCTCGAGTTA**CTTATCGTCGTCATCCTTGTAATC**TCCAATGACTTGACTGTATT |
| ERO1DR-F-SacII | CCGCCCGCGGATTAAAAAAAAAAAGACTAG |
| ERO1DR-R-SacI | GCCAGAGCTCTTGGAAGATGCCTAATGTGAA |
| HAC1-F | AGACGCTTTTTGGAATTACCCATCACCA |
| HAC1-R | TCAAAGTCCAACTGAAATG |
| SOD1-F | TGTTGTCAGAGGTGATTCAAAAGTC |
| SOD1-R | GTTGGAGCGGATTCGGATT |
| CAT1-F | ATTTCATCCACACCCAAAAGAGA |
| CAT1-R | CAAGTAATCCCAAAACATGTTAGCA |
| GPX2-F | TTGGTGTGACTTTCCCCGTATT |
| GPX2-R | CCGGGCTTTTGAGACTTCAA |
| PMT4-F | TTGCGTTACGAGGATGGAAGA |
| PMT4-R | TTTGGTGCCTTTGTTGGCTC |
| YSY6-F | AACACCTAAACAAAGAGCAGCTAA |
| YSY6-R | TGCTCCACCACATACTAAGAATAA |
| GAA1-F | TGGAATATTGTTCGCGGGTATA |
| GAA1-R | TCGGCCACACTCCACTCTTC |
| ERD2-F | TGCTCTTGGAATTTATCGTGCTT |
| ERD2-R | TCCTGCCAATACAGAAACAAAATC |

[a] Restriction sites introduced into the primer are underlined; the *FLAG* DNA sequence is in boldface.

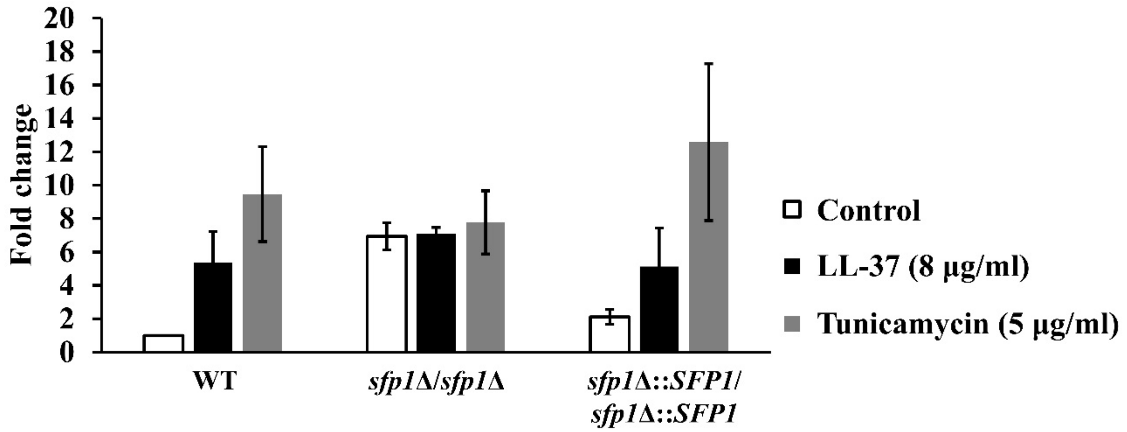


**Figure S1.** Integrated quantification of the three independent western blot analysis for p-Mkc1 detection. Cells were treated with or without LL-37 (8 µg/ml) or tunicamycin (5 µg/ml) for 30 min. Equal amounts of protein extracts from each sample were loaded. p-Mkc1 was monitored by western blotting and analyzed with ImageJ software. The Act1 band of each sample served as the loading control and was used to normalize the phosphor-Mkc1 levels. The fold change value of each sample is averaged from three independent western blot analysis.
